# Distinct expression of the neurotoxic microRNA family *let-7* in the cerebrospinal fluid of patients with Alzheimer's disease

**DOI:** 10.1371/journal.pone.0200602

**Published:** 2018-07-16

**Authors:** Katja Derkow, Rosa Rössling, Carola Schipke, Christina Krüger, Jakob Bauer, Michael Fähling, Andrea Stroux, Eckart Schott, Klemens Ruprecht, Oliver Peters, Seija Lehnardt

**Affiliations:** 1 Institute of Cell Biology and Neurobiology, Center for Anatomy, Charité - Universitaetsmedizin Berlin, Berlin, Germany; 2 Institute of Neuropathology, Charité - Universitaetsmedizin Berlin, Berlin, Germany; 3 Institute of Vegetative Physiology, Charité - Universitaetsmedizin Berlin, Berlin, Germany; 4 Institute for Biometry and Clinical Epidemiology, Charité - Universitaetsmedizin Berlin / Berlin Institute of health (BIH), Berlin, Germany; 5 Department of Hepatology and Gastroenterology, Charité - Universitaetsmedizin Berlin, Berlin, Germany; 6 Department of Neurology, Charité - Universitaetsmedizin Berlin, Berlin, Germany; 7 Department of Psychiatry, Charité - Universitaetsmedizin Berlin, Berlin, Germany; 8 German Center for Neurodegenerative Diseases (DZNE), Charité - Universitaetsmedizin Berlin, Berlin, Germany; 9 Cluster of Excellence NeuroCure, Charité - Universitaetsmedizin Berlin, Berlin, Germany; University of Washington, UNITED STATES

## Abstract

MicroRNAs (miRNAs) are non-coding RNAs originally involved in RNA silencing and post-transcriptional regulation of gene expression. We have shown in previous work that the miRNA let-7b can act as a signalling molecule for Toll-like receptor 7, thereby initiating innate immune pathways and apoptosis in the central nervous system. Here, we investigated whether different members of the miRNA family *let-7*, abundantly expressed in the brain, are released into the human cerebrospinal fluid (CSF) and whether quantitative differences in *let-7* copies exist in neurodegenerative diseases. RNA isolated from CSF of patients with Alzheimer´s disease (AD) and from control patients with frontotemporal lobe dementia (FTLD), major depressive episode (MDE) without clinical or neurobiological signs of AD, and healthy individuals, was reverse transcribed with primers against nine *let-7* family members, and miRNAs were quantified and analyzed comparatively by quantitative PCR. *let-7* miRNAs were present in CSF from patients with AD, FTLD, MDE, and healthy controls. However, the amount of individual *let-7* miRNAs in the CSF varied substantially. CSF from AD patients contained higher amounts of let-7b and let-7e compared to healthy controls, while no differences were observed regarding the other *let-7* miRNAs. No increase in let-7b and let-7e was detected in CSF from FTLD patients, while in CSF from MDE patients, let-7b and let-7e copy levels were elevated. In CSF from AD patients, let-7b and let-7e were associated with extracellular vesicles. *let-7* family members present in the CSF mediated neurotoxicity *in vitro*, albeit to a variable extent. Taken together, neurotoxic *let-7* miRNAs are differentially and specifically released in AD, but also in MDE patients. Thus, these miRNAs may mirror common neuropathological paths and by this serve to unscramble mechanisms of different neurodegenerative diseases.

## Introduction

Alzheimer´s disease (AD) is a progressive neurodegenerative disease and is considered to be the most common cause of dementia. Clinical symptoms of AD are characterized by deterioration of cognitive abilities due to hippocampal and whole brain atrophy [1]. Initial pathological changes in AD occur many years before clinical symptoms are apparent. Several pathogenic mechanisms in AD, such as a disturbed A-beta metabolism leading to amyloid plaque deposition accompanied by Tau-pathology, are known, and attempts have been made to bring them into a temporal order. Further, so far unknown key players are suggested to be involved in the neuropathological cascade of AD. Especially those playing the driving force for the progression of neurodegeneration are of interest.

MicroRNAs (miRNA) are non-coding RNAs that are involved in posttranscriptional regulation of gene expression. They bind to the 3´ untranslated regions of their target mRNAs, leading to repression of translation or to mRNA degradation. The human *let-7* miRNA family comprises nine mature members with strong sequence similarity, which are highly conserved across species [2]. Variation exists in the GU-rich sequences, but not in the seed sequence (see *Materials and Methods*). *Let-7* family members belong to the most abundantly expressed miRNAs in the brain [3]. miRNAs derived from various tissues are detectable in extracellular body fluids including cerebrospinal fluid (CSF) [4]. They are released from cells within exosomes [5] that may serve as intercellular signaling molecules within the brain, transferring their content to the respective target cells [6–8].

Given the miRNAs’ stability in body fluids and the possibility to detect low concentrations of these molecules by quantitative PCR, miRNAs make attractive targets in the search of novel biomarkers for neurodegenerative diseases. Variations in miRNA copy numbers in the CSF of patients with various CNS diseases, such as Alzheimer’s disease (AD) [9], traumatic brain injury [10], and HIV encephalitis [11] were reported. The miRNA let-7b acts as a ligand for toll-like receptor (TLR) 7 in microglia and neurons, thereby serving as an extracellular signaling molecule. Moreover, intrathecal let-7b mediates neurodegeneration in the CNS [12].

In this study, we systematically quantified members of the *let-7* family in the CSF of AD patients. For comparison, CSF from healthy controls and from patients with other CNS diseases associated with neurodegeneration and/or cognitive impairment such as frontotemporal lobe dementia (FTLD) and major depressive episode (MDE) [13] were analyzed.

## Materials and methods

### Clinical diagnosis and CSF sampling

The study was approved by institutional review boards (Ethikkommission Ethikausschuss 1 am Campus Charité-Mitte, Dementia competence network [14], EA1/182/10; BIH CRG 2a, EA2/118/15). Written informed consent was obtained from all patients participating in the study. CSF from patients with AD, FTLD, or MDE, and healthy controls were obtained from a local and a multicenter biomaterial bank. For patients diagnosed with AD, FTLD, or MDE neuropsychometric and biochemical characteristics as determined in the CSF were consistent with the diagnoses (Tables 1 and 2). Clinical diagnoses were determined in consensus conferences according to DSM-V, taking into account the patient’s history, neurological and psychiatric findings, neuropsychological test results, CSF biomarkers (t-Tau, Amyloid-β1–42) and cranial magnet resonance imaging. CSF cut-off values (Aβ1–42 ≤ 600 pg/ml, t-Tau ≥350 pg/ml) have previously been determined in-house performing ROC analyses on a data set comprising clinically validated diagnoses (80% for both sensitivity and specificity). To ensure reliability of biomarker values, CSF was collected and analyzed strictly according to protocols described elsewhere [15–17]. Briefly, lumbar puncture was performed with the patient in a sitting position. Exactly 12 ml CSF were collected in polypropylene tubes. Tubes were shaken, and CSF was centrifuged immediately after collection (1,600 *g*, room temperature, 10 min), aliquoted (250 μl), and frozen within 30 min after lumbar puncture. The material was stored at –80°C and was at no time thawed/refrozen. Sampling and storage conditions were identical for samples from all patient groups.

**Table 1:** Demographic data, neuropsychometric data, and biomarkers of patients analyzed in Fig 1, Fig 2, and Fig 3.

			Age		Total tau	Amyloid-β1–42
Patient ID	Diagnosis	Gender	(years)	MMSE^a^	(pg/mL)	(pg/mL)
AD-1	Alzheimer´s Disease	F	73	27	321	459
AD-2	Alzheimer´s Disease	F	66	24	344	570
AD-3	Alzheimer´s Disease	M	74	24	709	452
AD-4	Alzheimer´s Disease	F	73	27	646	573
AD-5	Alzheimer´s Disease	F	59	24	1,277	522
AD-6	Alzheimer´s Disease	F	54	24	368	396
AD-7	Alzheimer´s Disease	M	80	17	465	501
AD-8	Alzheimer´s Disease	F	70	12	535	673
AD-9	Alzheimer´s Disease	F	76	16	566	530
AD-10	Alzheimer´s Disease	F	78	22	297	364
AD-11	Alzheimer´s Disease	F	71	21	1,200	600
AD-12	Alzheimer´s Disease	F	84	25	659	645
**mean ± SD**	** **	** **	**71.5 ± 8.5**	**21.9 ± 4.6**	**615.6 ± 322.7**	**523.8 ± 94.9**
Co-1	Control	M	50	27	152	1,143
Co-2	Control	F	79	30	463	614
Co-3	Control	F	51	27	149	1,267
Co-4	Control	F	70	27	401	1,571
Co-5	Control	M	53	30	199	1,153
Co-6	Control	M	43	30	108	930
Co-7	Control	M	63	29	275	1,079
Co-8	Control	M	51	29	189	962
Co-9	Control	M	57	30	273	2,144
Co-10	Control	M	66	27	161	1,117
**mean ± SD**	** **	** **	**58.3 ± 11**	**28.6 ± 1.4**	**237 ± 116.4**	**1,198 ± 412.7**
FTLD-1	Frontotemporal lobe dementia	M	55	27	195	875
FTLD-2	Frontotemporal lobe dementia	F	75	22	452	1,222
FTLD-3	Frontotemporal lobe dementia	F	69	nd	567	793
FTLD-4	Frontotemporal lobe dementia	F	48	23	250	1,271
FTLD-5	Frontotemporal lobe dementia	M	61	27	493	1,953
FTLD-6	Frontotemporal lobe dementia	F	83	18	797	582
FTLD-7	Frontotemporal lobe dementia	F	65	15	412	1,088
FTLD-8	Frontotemporal lobe dementia	M	56	28	279	2,032
**mean ± SD**	** **	** **	**64 ± 11.5**	**22.9 ± 4.9**	**430.6 ± 195.5**	**1,227 ± 524.4**
MDE-1	Major depressive episode	M	51	23	132	867
MDE-2	Major depressive episode	F	60	28	361	1,440
MDE-3	Major depressive episode	M	62	27	290	825
MDE-4	Major depressive episode	F	78	26	428	820
MDE-5	Major depressive episode	M	49	30	221	1,067
MDE-6	Major depressive episode	F	48	26	106	716
MDE-7	Major depressive episode	F	64	13	254	1,670
MDE-8	Major depressive episode	F	52	24	194	1,067
**mean ± SD**	** **	** **	**58.0 ± 10.2**	**24.6 ± 5.2**	**248.3 ± 110.0**	**1,059 ± 334.9**

^a^MMSE: Mini-Mental State Examination

nd: not determined

**Table 2:** Demographic data, neuropsychometric data, and biomarkers of patients analyzed in Fig 4.

			Age		Total tau	Amyloid-β1–42
Patient ID	Diagnosis	Gender	(years)	MMSE^a^	(pg/mL)	(pg/mL)
AD-13	Alzheimer´s Disease	F	74	26	1,200	559
AD-14	Alzheimer´s Disease	F	70	25	479	406
AD-15	Alzheimer´s Disease	M	67	17	375	479
AD-16	Alzheimer´s Disease	F	67	23	1,020	444
AD-17	Alzheimer´s Disease	F	70	24	553	533
AD-18	Alzheimer´s Disease	F	76	18	761	558
AD-19	Alzheimer´s Disease	F	71	25	1,200	319
**mean ± SD**	** **	** **	**70.7 ± 3.4**	**22.57 ± 3.6**	**798.3 ± 345.1**	**471.1 ± 88.7**

^a^MMSE: Mini-Mental State Examination

nd: not determined

### RNA extraction from CSF

Total RNA was isolated using the *mir*Vana PARIS RNA Isolation kit (Life Technologies), as described previously [18]. Briefly, 400 μl of CSF were diluted with equal volumes of 2x denaturing solution before equal volumes of acid-phenol:chloroform were added. Samples were centrifuged at 15,000 *g* for 10 min. The aqueous phase was mixed with 1.25 volumes of 100% ethanol. After sample application to a filter cartridge, washing was performed according to the manufacturer's instructions. RNA was eluted in 100 μl of RNAse-free water. RNA was quantified by nanophotometry (Implen).

### MicroRNA reverse transcription and quantitation by real-time PCR

30–40 ng of total RNA of CSF samples from AD, FTLD, and MDE patients as well as controls were reverse transcribed using the TaqMan MicroRNA Reverse Transcription Kit (Applied Biosystems). In case of miRNA isolation from exosomes 5 μl RNA were used. MiRNA levels expressed as CT-values were determined by real time PCR (qPCR) using TaqMan MicroRNA assay for hsa-let-7a, hsa-let-7b, hsa-let-7c, hsa-let-7d, hsa-let-7e, hsa-let-7f, hsa-let-7g, hsa-let-7i, hsa-miR-98, hsa-miR-16, hsa-miR-24, and hsa-miR-124 according to the manufacturer's instructions on a 7500 Fast Real Time PCR system (Applied Biosystems). Expression levels of miRNAs in the CSF from one individual patient were determined in parallel on one single PCR plate. Quantitative miRNA levels expressed as absolute copy numbers were determined using TaqMan MicroRNA assay on a StepOnePlus Real Time PCR system (Applied Biosystems). Copy numbers were calculated implementing a standard curve using synthetic oligoribonucleotides and plotting CT-values versus copy number, a CT-value of 40 corresponding to a copy number of 0, as described previously [19]. qPCR was performed in triplicates. Subsequent evaluation was performed, as previously described [11]. One value was excluded from the triplicate if CT-values differed ≥1. In case all three values had a CT-difference ≥1 or at least two out of three replicas had a CT of 40 the respective miRNA was defined as not detected and was graphically depicted with CT 40.

### Isolation of extracellular vesicles from CSF

Isolation of extracellular vesicles from CSF was performed with the Total Exosome Isolation Kit according to the manufacturer’s instruction (Life Technologies). Briefly, 200 μl (for RNA isolation) or 400 μl (for western blotting) of CSF were thawed on ice and centrifuged at 10,000 *g* for 30 min at 4°C. One volume of exosome isolation reagent was added to the supernatant and mixed by vortexing before incubating for 1 h at 4°C. Samples were centrifuged at 10,000 *g* for 1 h at 4°C. Supernatant was aspirated. The complete volume of supernatant was used for RNA isolation. The pellet containing extracellular vesicles was re-suspended in either 200 μl PBS (for RNA isolation; Total Exosome RNA and Protein Isolation Kit, Life Technologies) or 20 μl ice-cold Exosome Resuspension Buffer (for western blotting).

### Western blotting

Western blotting was performed, as previously described [20]. Anti-Flotillin-1 and anti-CD63 antibodies (both at 1:250, BD Biosciences) were used.

### Oligoribonucleotides

The following native oligoribonucleotides were synthesized by Purimex DNA/RNA-Oligonucleotide, Grebenstein, Germany: let-7a, 5′-UGAGGUAGUAGGUUGUAUAGUU-3′; let-7b, 5′-UGAGGUAGUAGGUUGUGUGGUU-3′; let-7c, 5′ UGAGGUAGUAGGUUGUAUGGUU-3′; let-7d, 5′-AGAGGUAGUAGGUUGCAUAGUU-3′; let-7e, 5′-UGAGGUAGGA GGUUGUAUAGUU-3′; let-7f, 5′-UGAGGUAGUAGAUUGUAUAGUU-3′; let-7g, 5′-UGAGGUAGUAGUUUGUACAGUU-3′; let-7i, 5′-UGAGGUAGUAGUUUGUGCUGUU-3′; mutated oligoribonucleotide (mut. oligo), 5′-UGAGGUAGAAGGAUAUAAGGAU-3′.

### Primary neurons and cell lines

Cortical neurons were generated from C57BL/6 mice and cultured, as described previously [21]. SH-SY5Y neuroblastoma cells (ATCC) were cultured in DMEM supplemented with 10% heat-inactivated fetal calf serum (vol/vol) and 1% penicillin-streptomycin.

### *In vitro* miRNA toxicity assay

Various doses of oligoribonucleotides and other reagents were added to cell cultures for various durations, as indicated. Imiquimod and DAPI were obtained from InvivoGen and Roche, respectively. Terminal deoxynucleotidyl transferase-mediated biotinylated UTP nick end labeling (TUNEL) staining of cells was conducted using the In Situ Cell Death Detection kit, TMR red (Roche). For each condition, experiments were performed in duplicates.

### Immunocytochemistry

Immunostaining was performed, as described previously [20]. NeuN, neurofilament, and microtubule-associated protein 2 (MAP2) antibodies were used (all 1:1,000, Millipore). Alexa fluor 488 goat-anti-mouse and Alexa fluor 568 goat-anti-rabbit (both 1:500, Life Technologies) were used as secondary antibodies. Fluorescence microscopy was performed on an Olympus BX51 microscope.

### Quantitation of CNS cells *in vitro*

NeuN-positive cells were quantified by analyzing six high-power fields per coverslip. Numbers of NeuN-positive cells observed for each condition were compared with control conditions. Results were expressed as relative neuronal viability. TUNEL-positive cells were quantified in six high-power fields per coverslip and were expressed relatively to NeuN-positive cells in the same high power field as fold change to negative control.

### Flow cytometry

SH-SY5Y cells were fixed, permeabilized using the BD Cytofix/Cytoperm^TM^ Kit (BD Biosciences), and stained with anti-TLR7 Alexa488 antibody (Imgenex). An Alexa 488-conjugated isotype served as negative control. Data were collected on a BD FACSCanto II and analyzed by FlowJo Version 9.5.2.

### Statistical analysis

Data are expressed as indicated in the figures. Statistics as indicated in the figure legends were calculated using GraphPad Prism version 5.0 for Mac OS X (GraphPad Software, San Diego, USA). Otherwise, the statistical software SPSS 24 was used. In detail, data were described as mean or median and standard deviation. Statistical differences between two groups of individuals or patients were determined by the Student’s *t* test. Statistical differences over multiple groups of individuals or patients were determined using the one-way ANOVA test followed by Sidak’s multiple comparison post hoc test of patient group *vs*. control group. Adjusted *p*-values for patient and control groups were determined by ANCOVA with gender and age as covariates. Statistical differences over all tested conditions *in vitro* were determined using the Kruskal-Wallis test followed by Dunn´s Multiple comparison post hoc test of total *vs*. fraction or negative control vs. treatment. Differences were considered statistically significant when *p* < 0.05.

## Results

### Members of the *let-7* miRNA family are present in CSF of AD patients and healthy controls

**Fig 1:**
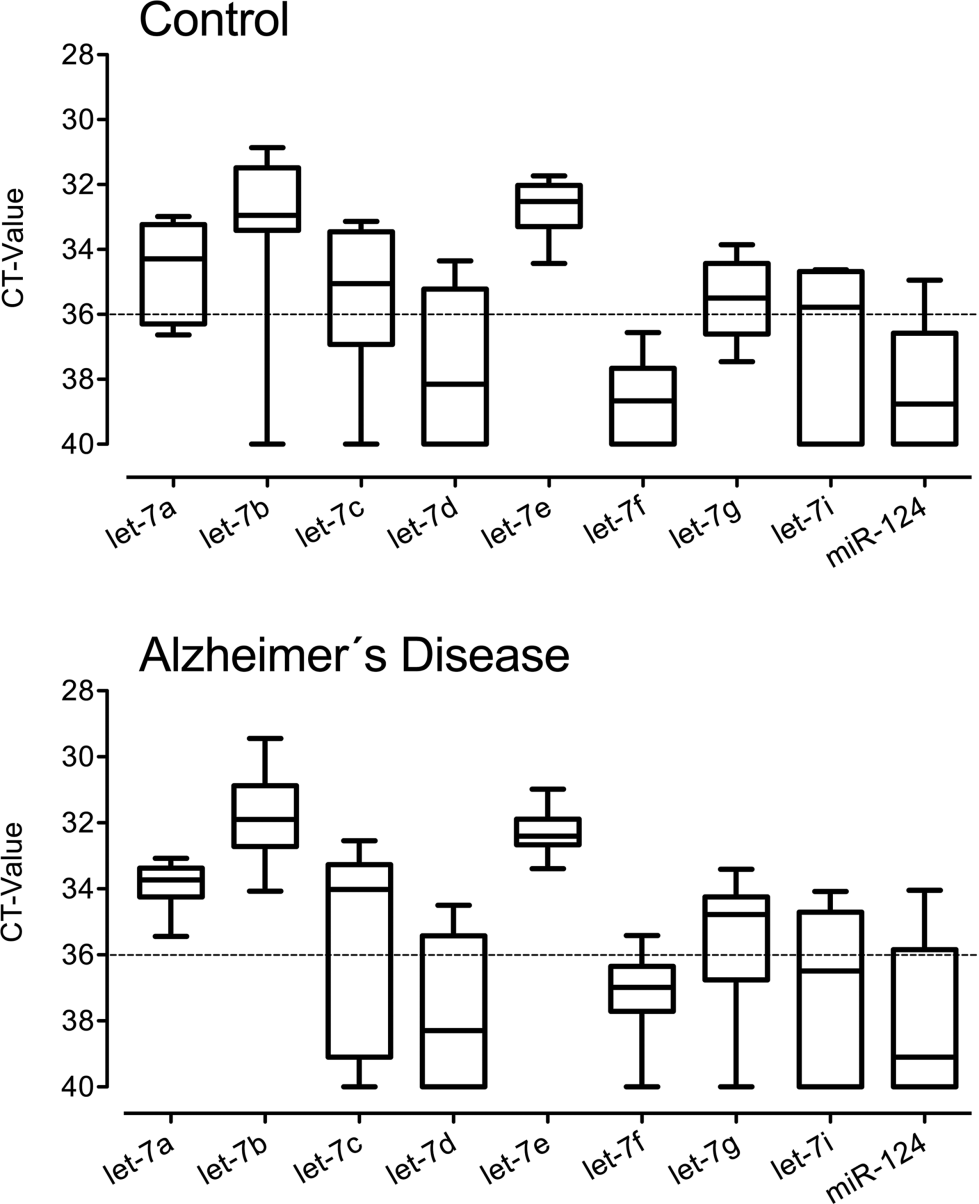
Members of the *let-7* miRNA family are present in CSF from healthy controls and patients with AD. CSF from healthy controls (*n* = 10, Co-1 –Co-10) or patients with AD (*n* = 12, patient AD-1 –AD-12) were assayed by qPCR using primers specific for let-7a, let-7b, let-7c, let-7d, let-7e, let-7f, let-7g, let-7i, or miR-124. Data are presented as box-and-whisker-plots of mean CT-values performed in triplicates with median for each miRNA.

Extracellular presence of let-7a, let-7b, let-7c, let-7d, let-7e, let-7f, let-7g, and let-7i in CSF from AD patients and healthy controls was analyzed by qPCR. In addition, the *let-7*-related miRNA miR-98, miR-124, which is abundantly and specifically expressed in the brain [22], as well as miR-16 and miR-24, which both were previously described as reference miRNAs in CSF [18, 23], were included in our study. Results were expressed as median CT-values. While *let-7* miRNAs were present at different copy levels in different individuals, expression patterns were similar in AD patients and healthy controls. In particular, let-7a, let-7b, let-7c, and let-7e were found at high copy levels, while let-7d, let-7f, let-7g, let-7i, and miR-124 were present at lower copy levels in the majority of samples or were only marginally detected (determined as CT-values >36) in both experimental groups (Fig 1, Table 3). miR-98, miR-16, and miR-24 were not reliably detectable in CSF from both groups (S1 Fig).

**Table 3:** Median CT-values from data depicted in Fig 1 and Fig 3A.

Diagnosis	let-7a	let-7b	let-7c	let-7d	let-7e	let-7f	let-7g	let-7i	miR-124
									
Control	34.30	32.96	35.06	38.16	32.53	38.68	35.51	35.79	38.77
Alzheimer´s Disease	33.74	31.91	34.03	38.31	32.42	36.99	34.78	36.50	39.11
Frontotemporal lobe dementia	33.47	32.37	34.50	40	32.63	37.15	34.97	35.67	32.51
Major depressive episode	33.61	31.66	34.11	35.30	31.83	37.28	33.99	35.31	37.84

### CSF from AD patients contains high amounts of let-7b and let-7e

**Fig 2:**
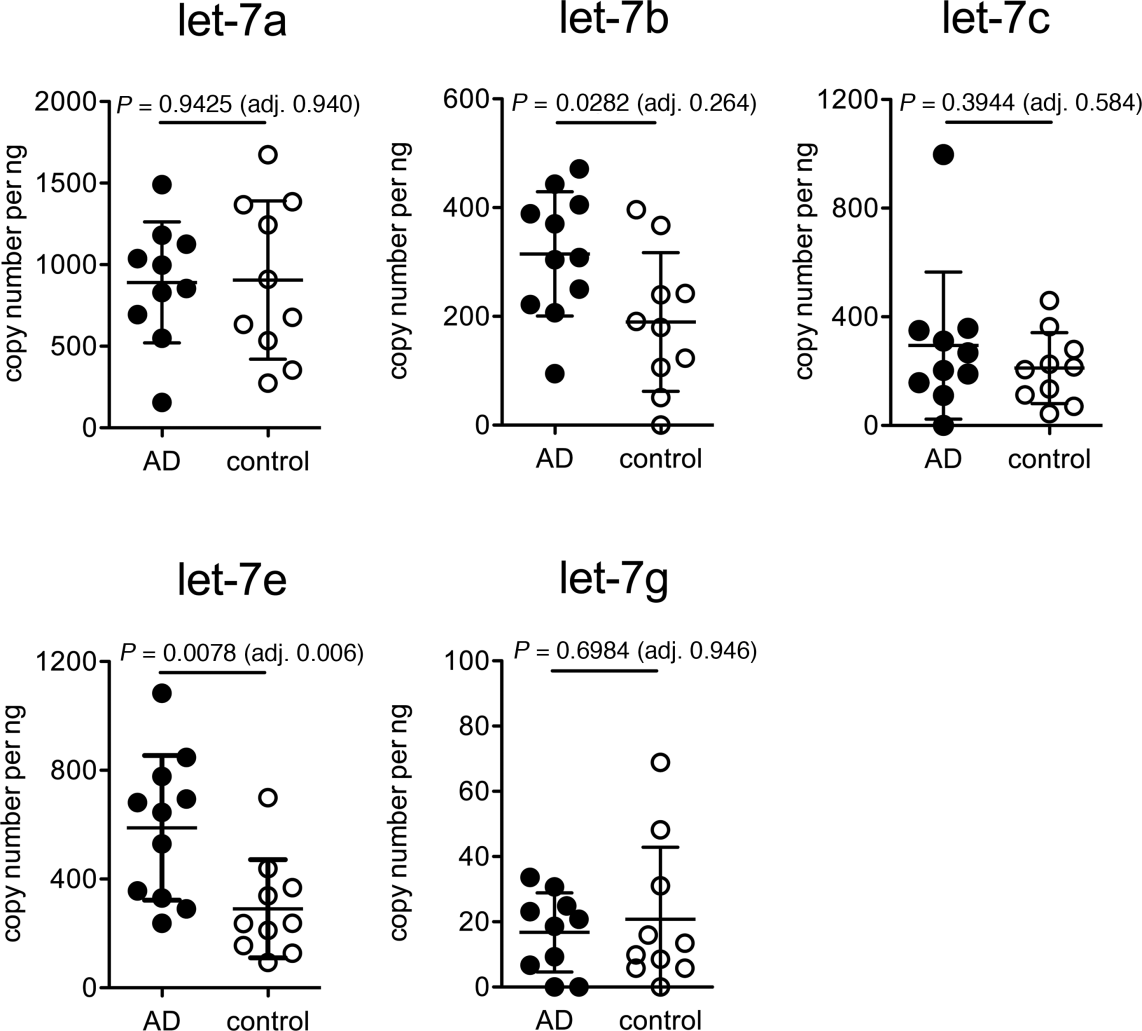
CSF from patients with AD contains elevated levels of let-7b and let-7e copies compared to healthy controls. CSF from patients with AD (*n* = 10–11, AD-1 –AD-6; AD-8 –AD-12) and healthy controls (*n* = 10, Co-1 –Co-10) were assayed by qPCR using primers specific for let-7a, let-7b, let-7c, let-7e, or let-7g and were normalized to the standard of the respective synthetic miRNA. Data are presented as mean ± SD. Each patient resembles one dot. Statistical analysis was performed using unpaired *t*-test, and *p*-values were adjusted (adj.) for age and gender by ANCOVA, as indicated.

While the presence of different *let-7* miRNAs within the CSF from one patient or healthy individual was determined by analyzing raw median CT-values from qPCR runs in the approach described above, in a second step we aimed at determining and comparing the absolute quantities of the different *let-7* miRNAs specifically present in CSF from AD patients and from healthy controls. To quantitate the copy number of individual miRNAs in CSF analyzed by qPCR, values were normalized to a standard dosage of the respective synthetic oligoribonucleotide. For direct quantitative comparison, samples of AD patients and controls were assayed on one plate within one PCR run. Only those *let-7* miRNAs were quantitated that reached the defined cut-off of median CT-values <36 in the CSF from AD patients and controls tested in the experiments described above, namely let-7a, let-7b, let-7c, let-7e, and let-7g (Fig 2, S2 Fig). CSF from AD patients contained significantly elevated copy levels of let-7b and let-7e as determined by *t*-test and, if adjusted for gender and age, of let-7e only compared to controls. In contrast, copy numbers of let-7a, let-7c, and let-7g did not differ between AD CSF and control CSF, indicating a selective alteration of let-7b and let-7e copies in the CSF from AD patients.

### Presence of *let-7* miRNAs in CSF from FTLD and MDE patients

**Fig 3:**
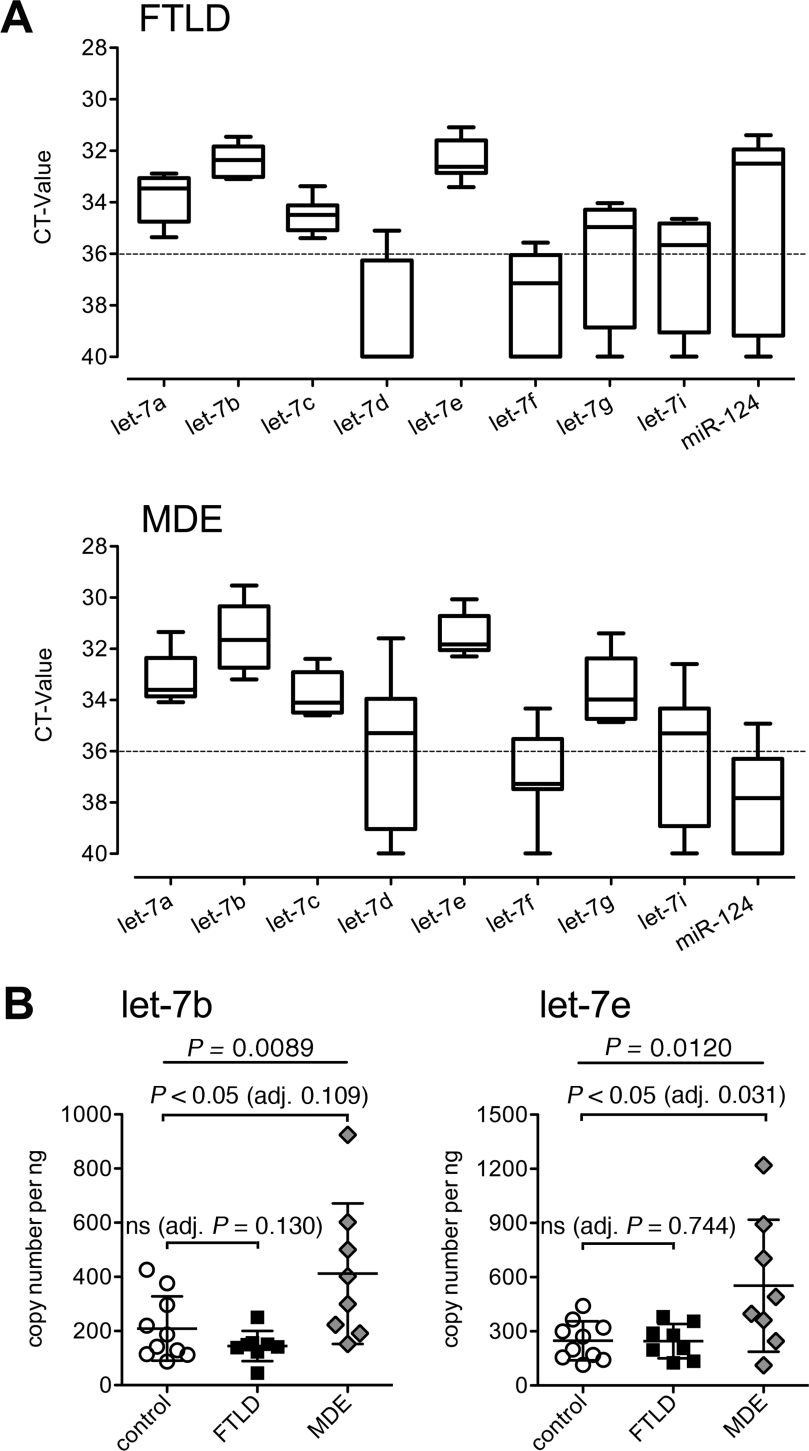
CSF from patients with MDE, but not with FTLD, contains elevated levels of let-7b and let-7e copies compared to healthy controls. (A) CSF from healthy controls (*n* = 10, Co-1 –Co-10) and from patients with FTLD (*n* = 8, FTLD-1 –FTLD-8) or MDE (*n* = 8, D-1 –D-8) were assayed by qPCR using primers specific for let-7a, let-7b, let-7c, let-7d, let-7e, let-7f, let-7g, let-7i, or miR-124. Data are presented as box-and-whisker-plots of mean CT-values performed in triplicates with median for each miRNA. (B) CSF from samples described in (A) were assayed by qPCR using primers specific for let-7b or let-7e and were normalized to the standard of the respective synthetic miRNA. Data are presented as mean ± SD. Each patient resembles one dot. Statistical analysis was performed using one-way ANOVA followed by Sidak’s multiple comparison post hoc test of the respective patient group *vs*. control group. Global *p*-values, as indicated in the figure. ns, not significant. *p*-values were adjusted (adj.) for age and gender by ANCOVA, as indicated.

Having identified a specific expression pattern of *let-7* miRNAs in the CSF from AD patients, we investigated whether CSF from patients with other CNS conditions associated with neurodegeneration and/or cognitive impairment likewise contains *let-7* miRNAs. To this end, CSF from FTLD and MDE patients without clinical or neurobiological signs of AD served as disease controls (see Table 1 for patient details) and were tested for the presence of *let-7* miRNAs using qPCR. All tested *let-7* miRNAs and miR-124 were detected at different expression levels within one patient of both groups (Fig 3A). Expression patterns of *let-7* miRNAs in the CSF from individual FTLD and MDE patients were similar and resembled those observed in AD patients. In both CSF from FTLD and MDE patients median CT-values for let-7a, let-7b, let-7c, let-7e, let-7g, and let-7i were below the threshold of CT 36, but median CT-values for let-7f were above CT 36. let-7d was present in 7 out of 8 samples from MDE patients (median CT-value 35.30), while it was detected in only 3 out of 8 CSF samples from FTLD patients. For comparison, let-7d was present in 6 out of 12 CSF samples from AD patients and in 3 out of 10 from healthy controls. CT-values for miR-124 expression were elevated in CSF from FTLD patients compared to all other tested groups (detectable in 6 out of 8 samples from FTLD patients, median CT-value 32.51, see Table 3).

To quantify those two *let-7* miRNAs being present at high expression levels in AD, namely let-7b and let-7e, in CSF from FTLD and MDE patients, absolute copy numbers of both miRNAs were determined by qPCR (Fig 3B, S3 Fig). In this experiment, CSF from the same control individuals described above was analyzed. No significant variation between the values derived from the different runs with control CSF samples (Fig 2 *vs*. Fig 3B) was detected (S4 Fig). In contrast to our findings in CSF from AD patients let-7b and let-7e copy numbers did not differ between the FTLD CSF and the control CSF (Fig 3B, S3 Fig). However, compared to controls copy numbers of let-7b and let-7e, as determined by *t*-test, and let-7e only, if adjusted for age and gender, were elevated in CSF from MDE patients. A-beta values were normal in these patients (see Table 1).

### let-7b and let-7e are enriched in extracellular vesicles of AD CSF

**Fig 4:**
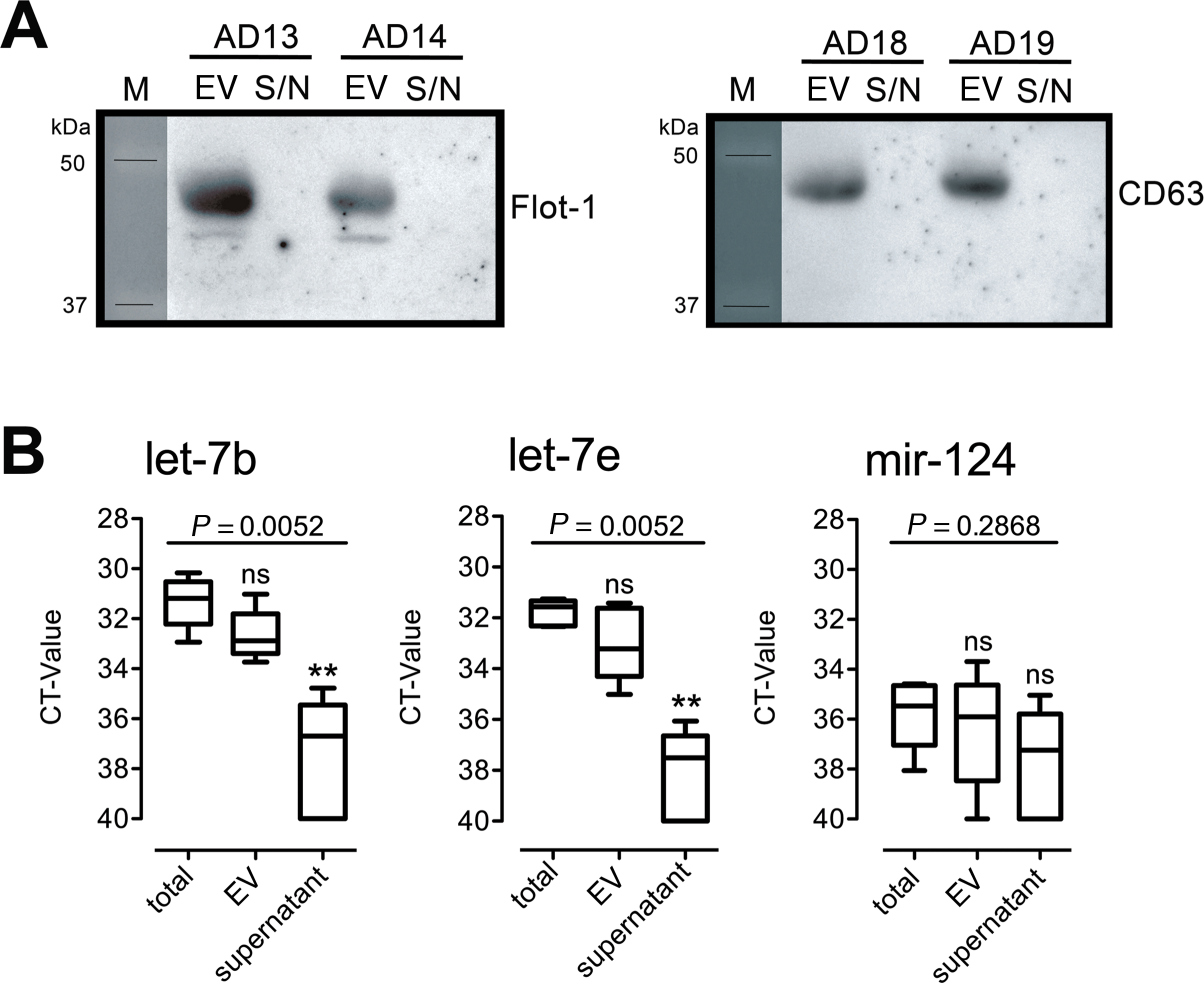
let-7b and let-7e are enriched in the fraction of extracellular vesicles in the CSF from AD patients. (A) Extracellular vesicles (EV) were isolated from CSF of AD patients (*n* = 4), and both pellet of EV and supernatant obtained during the isolation procedure were subjected to western blot using flotillin-1 or CD63 Abs, which served as exosome-specific markers. (B) RNA was isolated from whole CSF (total) and from both the pellet of EV and supernatant derived from the same AD patient (*n* = 5, AD-15 –AD-19). Samples were assayed by qPCR using primers specific for let-7b, let-7e, or miR-124. Data are presented as box-and-whisker-plots of mean CT-values performed in triplicates with median for each miRNA. Statistical analysis was performed using the Kruskal-Wallis test followed by Dunn´s Multiple comparison post hoc test of total *vs*. fraction, **p<0.01; ns, not significant. Global *p*-values are indicated.

To investigate whether *let-7* miRNAs in human CSF are associated with extracellular vesicles, extracellular vesicles were isolated from CSF of selected AD patients (for patient characteristics see Table 2). Copy levels of let-7b and let-7e that were specifically expressed in AD CSF in large quantities were determined in whole CSF (total), in the fraction of extracellular vesicles, and in the supernatant devoid of extracellular vesicles by qPCR. miR-124 served as a control for miRNA specificity (Fig 4). Successful isolation of extracellular vesicles including exosomes from CSF was confirmed by western blot using flotillin-1 and CD63 antibodies that represent exosome markers [24, 25] (Fig 4A, for whole blot images see S5 Fig). CT-values obtained for let-7b and let-7e in miRNA preparations from extracellular vesicles of AD CSF did not differ from CT-values detected in miRNA preparations from whole CSF. miRNA copy levels in the CSF supernatant were significantly lower compared to both whole CSF and extracellular vesicles, or were not detectable (Fig 4B), indicating an association of let-7b and let-7e with extracellular vesicles in the CSF. miR-124 was only marginally present in whole CSF and was not detected in the fraction of extracellular vesicles or supernatant fraction.

### Extracellularly delivered let-7b and let-7e mediate neuronal injury *in vitro*

**Fig 5:**
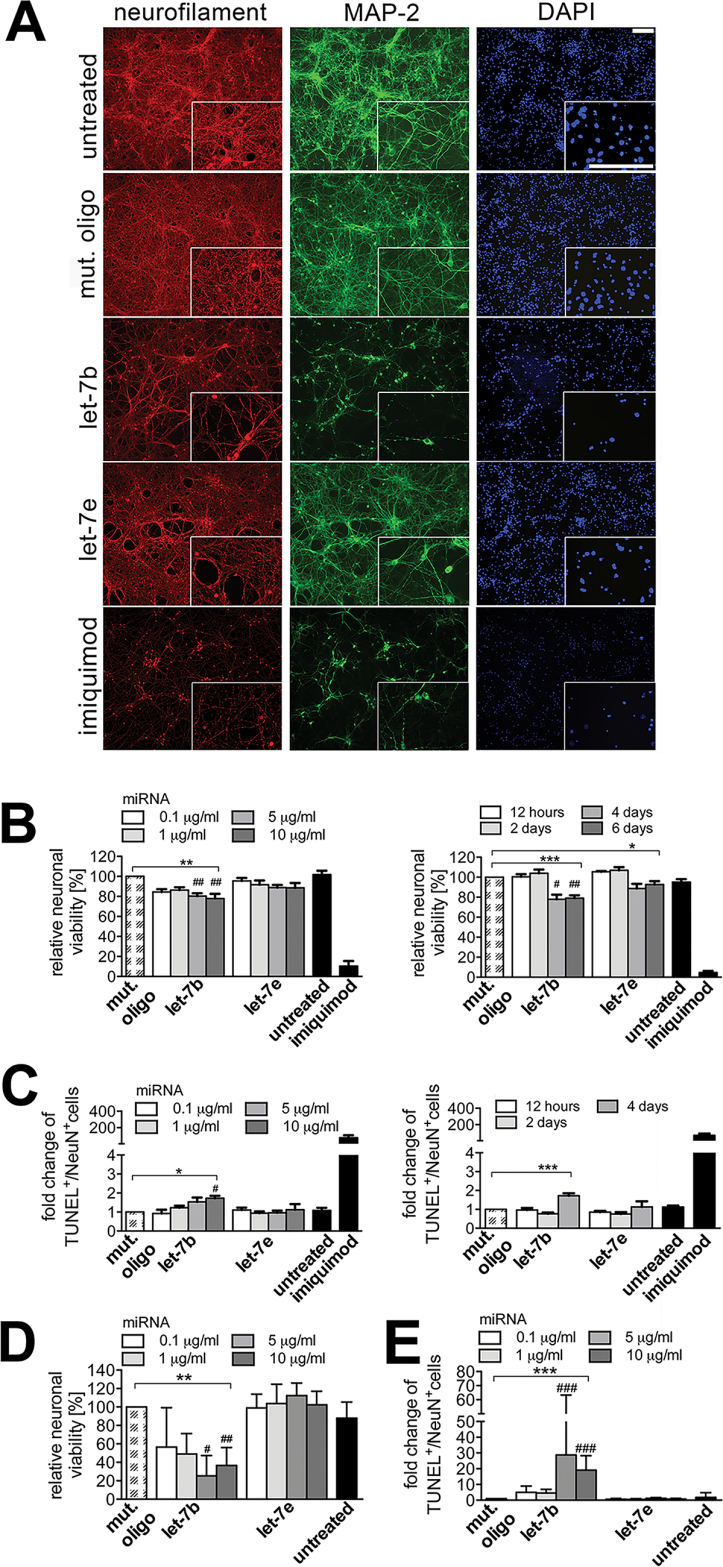
Neurotoxic potential of let-7b and let-7e *in vitro*. (A) Cortical neurons from C57BL/6 mice were incubated with 5 μg/ml of the respective native oligoribonucleotide, as indicated, for 5 days. Treatment with imiquimod (10 μg/ml) served as a positive control. Subsequently, cells were fixed and immunostained with neurofilament Ab (red), MAP-2 Ab (green), and stained with DAPI (blue). Scale bar 100 μm. (B, C) C57BL/6 neurons or (D, E) SH-SY5Y cells were incubated with indicated doses of native let-7b, let-7e, or a mutated control oligoribonucleotide for 4 days or with 5 μg/ml of native let-7b, let-7e, or a mutated control oligoribonucleotide for indicated durations. Treatment with imiquimod (10 μg/ml) served as a positive control. Cells were immunostained with NeuN Ab, stained by TUNEL assay, or with DAPI. Each condition was performed in duplicate and averaged. NeuN-positive cells were quantified and were expressed as relative neuronal viability. TUNEL-positive cells were quantified, set in relation to NeuN-positive cells, and were expressed as fold-change. Mean ± SD from 3–4 individual experiments, Kruskal-Wallis test followed by Dunn´s Multiple comparison post hoc test of negative control *vs*. treatment. *P* values in (B) depicting the dose response: let-7b **p = 0.0036; time response: let-7b ***p = 0.0002, let-7e *p = 0.0107. *P* values in (C) depicting the dose response: let-7b *p = 0.0136; time response: let-7b ***p = 0.0005. (D, E) *P*-values for relative neuronal viability: let-7b **p = 0.0047; TUNEL assay: let-7b ***p = 0.0001. #*p*<0.05, ##*p*<0.01, ###*p*<0.005. mut. oligo, mutated oligoribonucleotide.

To investigate the neurodegenerative potential of let-7b and let-7e, murine cortical neurons were incubated with chemically unmodified let-7b, let-7e, or a mutated oligoribonucleotide, which served as a negative control. Subsequently, cellular integrity and relative neuronal viability were analyzed by immunocytochemistry. In response to let-7b pronounced axonal and dendritic injury compared to control conditions were observed. Although to a much lesser extent, let-7e also induced injury of axons and dendrites (Fig 5A). Assessment of the relative neuronal viability revealed that let-7b led to significant loss of neurons dose- and time-dependently. In detail, incubation with let-7b reduced neuronal viability to 79.03% after 6 days compared to control conditions. Incubation with let-7e led to a reduced neuronal survival over time but did not result in dose-dependent effects (Fig 5B). Other *let-7* miRNA family members also mediated neuronal injury *in vitro*. In detail, let-7f and let-7g, and to a lesser extent let-7a, let-7c, let-7d, and let-7i induced axonal and dendritic injury (Part A of S6 Fig). let-7f and let-7g induced significant loss of neurons dose- and time-dependently, whereas let-7a, let-7c, let-7d, and let-7i did not mediate neuronal loss (Part B of S6 Fig). Analysis of neurons by TUNEL assay revealed increased apoptosis in response to let-7b, let-7f, and let-7g. In contrast, treatment with let-7c, let-7d, and let-7e did not result in a significant increase in TUNEL-positive neurons (Fig 5C, Part C, D of S6 Fig).

To confirm the injurious effects of *let-7* miRNAs on human-derived neurons and to ensure that the observed degenerative effects were cell-autonomous and not due to contamination with other cells, e.g. microglia, human neuroblastoma SH-SY5Y cells, which express TLR7 (S7 Fig), were incubated with various doses of let-7b and let-7e that were present at high copy levels in AD and MDE CSF (Fig 5D and 5E). let-7b, but not let-7e induced significant loss of neurons dose-dependently (Fig 5D). Furthermore, induction of apoptosis in response to let-7b, but not to let-7e was observed (Fig 5E).

## Discussion

We systematically investigated the expression of *let-7* miRNAs in the CSF of patients with AD, FTLD, MDE, and healthy individuals. All tested *let-7* miRNAs were detected, although in different quantities. In particular, copy numbers of let-7b and let-7e were increased in AD and MDE, but not in FTLD patients. The selective increase in let-7b and let-7e in AD and MDE may reflect a common pathway of neurodegeneration in these diseases [13].

We showed in previous work that intrathecal let-7b, preferentially expressed in neurons and released from injured cells, induces further neurodegeneration via endolysosomal TLR7, a GU-rich sequence motif within the oligoribonucleotide being essential for receptor activation [12]. The different *let-7* miRNAs share sequence similarity and contain highly conserved, but not identical GU-rich 3´ sequences. In the study named above, incubation of cultured neurons with up to 10 μg/ml of the oligoribonucleotide caused significant neuronal loss [12]. Based on these observations we set out by systematically analyzing the ability of different extracellularly delivered *let-7* miRNAs to induce neurotoxicity *in vitro*. All tested *let-7* miRNAs mediated neuronal injury, although to different extents. These effects were mediated by oligoribonucleotides without chemical modification, indicating a sufficient stability of these molecules and a distinct biological activity in their extracellular form under pathophysiological conditions. However, it is unclear at this stage how much *let-7* is effectively released at the site of brain injury in neurodegenerative disorders and what the local concentration maxima are. Our study is a proof of principle regarding the effects of *let-7* miRNAs in the brain, and therefore, supraphysiological doses were used. Future work will be required to determine the pathophysiological concentrations of the different *let-7* miRNAs and their neurotoxic potential in detail *in vivo*. Finally, although we have shown previously that extracellularly delivered let-7b induces neuronal caspase-3 via TLR7 activation [12], and some of the tested *let-7* miRNAs mediated apoptosis and were detected within CSF-derived extracellular vesicles in the current study, the exact signalling pathway linking the canonical TLR pathway and neuronal apoptosis remains to be elucidated. Also, the exact nature of the CSF-associated extracellular vesicles is unclear since most strategies used for the preparation of extracellular vesicles including the method used in this study (see *Materials and Methods*) cannot reliably distinguish between different specific extracellular vesicle species such as exosomes or between extracellular vesicles and protein aggregates, such as protein-miRNA complexes [26]. Nevertheless, we speculate that *let-7* miRNAs bind to TLR7 only when associated with extracellular vesicles that are released during neuronal injury. Thereby, these oligonucleotides may engage TLR7 in the endolysosomal compartment of neighboring neurons and mediate injury, whereas cytosolic miRNAs do not mediate injury via TLR7 in intact neurons.

In this study expression levels of miRNAs in human CSF were determined for one individual patient on one PCR plate to compare copy levels of different miRNAs within one AD, FTLD, or MDE patient or within one control subject. Similar expression patterns of different *let-7* miRNAs were detected in the CSF from all patient groups. In particular, let-7b and let-7e were consistently present at high expression levels compared to the other miRNAs. Even if statistical analysis was adjusted for gender and age, let-7e was significantly elevated in AD and MDE CSF. In terms of let-7b, adjusted statistical testing pointed to a potential interaction between gender and AD, provided that additional male individuals are included in the study. In contrast to let-7b that induced profound neurodegeneration *in vitro*, let-7e mediated only mild neuronal injury. Likewise, let-7a and let-7c were detected at high copy levels in CSF from patients and controls, but exhibited only minor neurotoxic effects. Conversely, let-7f and let-7g mediated distinct neurodegeneration *in vitro* but copy numbers were low in all CSF samples. Thus, the capacity of inducing neurotoxicity *in vitro* did correlate with high copy numbers in the CSF in the case of let-7b, but this relation was not observed for the other *let-7* miRNAs. These findings may reflect distinct biological functions and potential of each *let-7* miRNA in its extracellular form.

While RNA quantities isolated from CSF did not relevantly differ between AD patients and controls, it did vary between controls and FTLD as well as MDE patients. Therefore, equal RNA amounts were applied for reverse transcription to standardize the experimental procedure. We determined copy numbers of the selected miRNAs by utilizing dosage curves of the respective synthetic miRNA and compared miRNA expression levels in the CSF with concentrations of the respective synthetic oligoribonucleotide. We report here on an increase of let-7b copies in the CSF from AD patients, thereby confirming our previous studies in which different patient cohorts were used [12]. In addition, absolute quantification of let-7b and let-7e revealed increased copy numbers in AD and MDE CSF, but not in FTLD CSF. Likewise, CSF from patients with clinically isolated syndrome, i.e. the first clinical manifestation of multiple sclerosis, which can be accompanied by cognitive impairment [27, 28], contains higher levels of let-7e copies compared to controls (S. Lehnardt, unpublished observation). let-7d, which was detected in the CSF from only few AD, FTLD, and control individuals, was consistently found at high copy levels in MDE CSF. Likewise, miR-124 was detected more reliably in FTLD CSF compared to other tested groups. Thus, miRNAs are differentially and specifically released into the CSF during the course of CNS diseases.

A previous study reported on increased let-7f amounts in AD CSF [9]. In the present study analysis of let-7f expression yielded in CT-values >36 in most CSF samples, independently on the respective disease context, and was therefore not further quantified. However, although only low levels of let-7f copies were detected in 10 out of 11 samples (91%) in AD CSF, in CSF from controls let-7f was present in only 6 out of 10 samples (60%), matching—at least in part—Cogswell´s observations. Additional steps of pre-amplification may be necessary to determine more reliable differences between miRNA copy numbers in CSF from patient and control groups. Also, interferences of CSF analysis with cellular and/or blood contamination have to be considered [29]. In our study, CSF probes were freed of cells by a standardized centrifugation protocol immediately after lumbar puncture. Thus, contamination of the samples with cells was not likely to affect our expression analysis. Variations between different studies regarding miRNA expression in CSF are not surprising considering the fact that various technical routines were applied. Therefore, a comparison regarding miRNA expression between different studies remains difficult at this stage. Further research is required to standardize the respective approaches.

The fact that only selected *let-7* miRNAs were detectable in sufficient amounts in the AD CSF may be due to specific packaging of miRNAs into extracellular vesicles such as exosomes and release into the CSF. miRNAs, which may be protected by their exosomal carriers, feature a major stability in CSF and are therefore considered as potential biomarkers for CNS diseases [18]. In the light of such potential usage we evaluated the stability of *let-7* miRNAs in AD CSF, which had been stored for extended periods (85,1+/-29,4 months after lumbar puncture). qPCR analysis revealed slightly reduced, but readily detectable miRNA copy levels (S8 Fig).

Although group sizes in our study were small, degrees of data variation were low and reliability was sufficient. However, we did not observe a significant correlation between amounts of amyloid-β1–42 or total-tau and let-7b or let-7e expression in the CSF. Furthermore, gender and age of the tested subjects were not evenly distributed among the groups due to random selection of biological samples from the biomaterial bank. There was neither a general gender bias in our biomaterial bank nor any gender bias in either of the diseases. Age differences between the tested groups are at least mostly of inherent nature since the biological samples were from observational studies, and study participants were recruited from a population referred to the memory clinic by general practioners. Depressed patients tend to be younger than AD patients in this kind of population. Furthermore, in average the onset of AD is substantially later than the onset of FTLD. Therefore, the comparison between patients with AD, FTLD or depression is challenged by age difference *per se*. Nevertheless, age and gender may influence the pattern of circulating miRNAs [30], and we cannot exclude that these parameters may have an effect on the expression of *let-7* miRNAs in the CSF in our study. Thus, to increase reliability, to determine whether expression levels of a specific *let-7* miRNA correlate with different disease stages or disease progression, and to carefully analyze individual aspects such as age and gender, future studies should consider extended and differentiated patient cohorts and determine exact *let-7* miRNA expression levels in combination with other diagnostic strategies including assessment of cognitive function, detection of classical markers of neurodegeneration, and neuroimaging.

In summary, *let-7* miRNAs that reveal neurotoxic properties *in vitro* are differentially and specifically released in AD. Given that quantities of *let-7* miRNAs in human CSF differ depending on a specific CNS disease context, that *let-7* miRNAs are stable in CSF and that their stability may greatly facilitate the standardization of sampling and detection procedures, these miRNAs may be attractive targets in the future search for new diagnostic and therapeutic strategies in neurodegenerative diseases.

## Supporting information

S1 Fig Expression levels of miR-98, miR-24, and miR-16 in CSF from healthy controls and patients with AD. CSF samples from healthy controls (*n* = 9, Co-2 –Co-10) or patients with AD (*n* = 10, patient AD-3 –AD-12) were assayed by qPCR using primers specific for miR-98, miR-24, or miR-16. Data are presented as box-and-whisker-plots of mean CT-values performed in triplicates with median for each miRNA.
(TIF)

S2 Fig Data determined in the analysis of *let-7* miRNA presence in AD and control CSF depicted in Fig 2. CSF from patients with AD (*n* = 10–11, AD-1 –AD-6; AD-8 –AD-12) and healthy controls (*n* = 10, Co-1 –Co-10) were assayed by qPCR using primers specific for let-7a, let-7b, let-7c, let-7e, or let-7g and were normalized to the standard of the respective synthetic miRNA. Statistical analysis was performed using unpaired *t*-test, and *p*-values were adjusted for age and gender by ANCOVA.
(DOCX)

S3 Fig Data determined in the analysis of *let-7b* and *let-7e* presence in FTLD, MDE and control CSF depicted in Fig 3B. CSF from healthy controls (*n* = 10, Co-1 –Co-10) and from patients with FTLD (*n* = 8, FTLD-1 –FTLD-8) or MDE (*n* = 8, D-1 –D-8) were assayed by qPCR using primers specific for let-7b or let-7e and were normalized to the standard of the respective synthetic miRNA. Statistical analysis was performed using one-way ANOVA followed by Sidak’s multiple comparison post hoc test of the respective patient group *vs*. control group. *p*-values were adjusted for age and gender by ANCOVA.
(DOCX)

S4 Fig Analysis of qPCR run-to-run variation in CSF of healthy controls. CSF from healthy controls depicted in Fig 2 and Fig 3B (*n* = 10, Co-1 –Co-10) were assayed by qPCR using primers specific for let-7b or let-7e and were normalized to the standard of the respective synthetic miRNA. Data are presented as mean ± SD. Each patient resembles one dot. Statistical analysis was performed using paired Student’s *t*-test.
(TIF)

S5 Fig Whole western blots of blot images depicted in Fig 4. Extracellular vesicles (EV) were isolated from CSF of AD patients (*n* = 4), and both pellet of EV and supernatant (S/N) obtained during the isolation procedure were subjected to western blot using flotillin-1 (A) or CD63 (B) Abs. Neurons: Cortical neurons isolated from C57BL/6J mice.
(TIF)

S6 Fig Extracellularly delivered *let-7* miRNAs induce neuronal injury *in vitro*. (A) Neurons from C57BL/6 mice were incubated with 5 μg/ml of the respective oligoribonucleotide or with 5 μg/ml imiquimod, which served as a positive control for TLR7-mediated neuronal cell death, for 5 days. Subsequently, cells were fixed and immunostained with neurofilament Ab (red), MAP-2 Ab (green), and stained with DAPI (blue). Scale bar 100 μm. (B, C) Neurons from C57BL/6 mice were incubated with indicated concentrations of let-7a, let-7c, let-7d, let-7f, let-7g, let-7i, or a mutated control oligoribonucleotide for 4 days, or with 10 μg/ml of the respective miRNA for indicated time points. 10 μg/ml imiquimod served as a positive control. Cells were immunostained with NeuN Ab, stained by TUNEL assay, or with DAPI. Each condition was performed in duplicate and averaged. NeuN-positive cells were quantified and were expressed as relative neuronal viability. TUNEL-positive cells were quantified, set in relation to NeuN-positive cells, and were expressed as fold-change. Mean ± SD from 3–4 individual experiments, Kruskal-Wallis test followed by Dunn´s Multiple comparison post hoc test of negative control *vs*. treatment. *P* values in (B) depicting the dose response: let-7f **p = 0.0033, let-7g **p = 0.0018; time response: let-7f **p = 0.0011, let-7g ***p = 0.0005. *P* values in (C) depicting the dose response: let-7f *p = 0.0223, let-7g **p = 0.0042; time response: let-7a ***p = 0.0008, let-7f ***p = 0.0009, let-7g ***p = 0.0005. #*p*<0.05, ##*p*<0.01, ###*p*<0.005. mut. oligo, mutated oligoribonucleotide. (D) Neurons were incubated with 10 μg/ml of the respective miRNA for 4 days. Subsequently, they were immunostained with NeuN Ab (green) and stained by TUNEL assay (red) and with DAPI (blue). Scale bar 50 μm.
(TIF)

S7 Fig TLR7 is expressed in SH-SY5Y cells. SH-SY5Y cells were fixed, permeabilized, stained with anti-TLR7 Alexa 488 Ab, and analyzed by flow cytometry. An Alexa488-conjugated isotype was used as a negative control.
(TIF)

S8 Fig Detection of *let-7* miRNAs in AD CSF that had been stored for extended periods of time. CSF samples from patients with AD (*n* = 10) that had been stored for 85,1+/-29,4 (mean ± SD) months after lumbar puncture at -80°C, were assayed by qPCR using primers specific for let-7b, let-7e, or miR-124. Data are presented as box-and-whisker-plots of mean CT-values performed in triplicates with median for each miRNA.
(TIF)
