# Supplementary figures and images for: Distinct expression of the neurotoxic microRNA family let-7 in the cerebrospinal fluid of patients with Alzheimer's disease
Source: PLoS One. 2018 Jul 16;13(7):e0200602. doi: 10.1371/journal.pone.0200602 (PMC6047809; doi:10.1371/journal.pone.0200602)

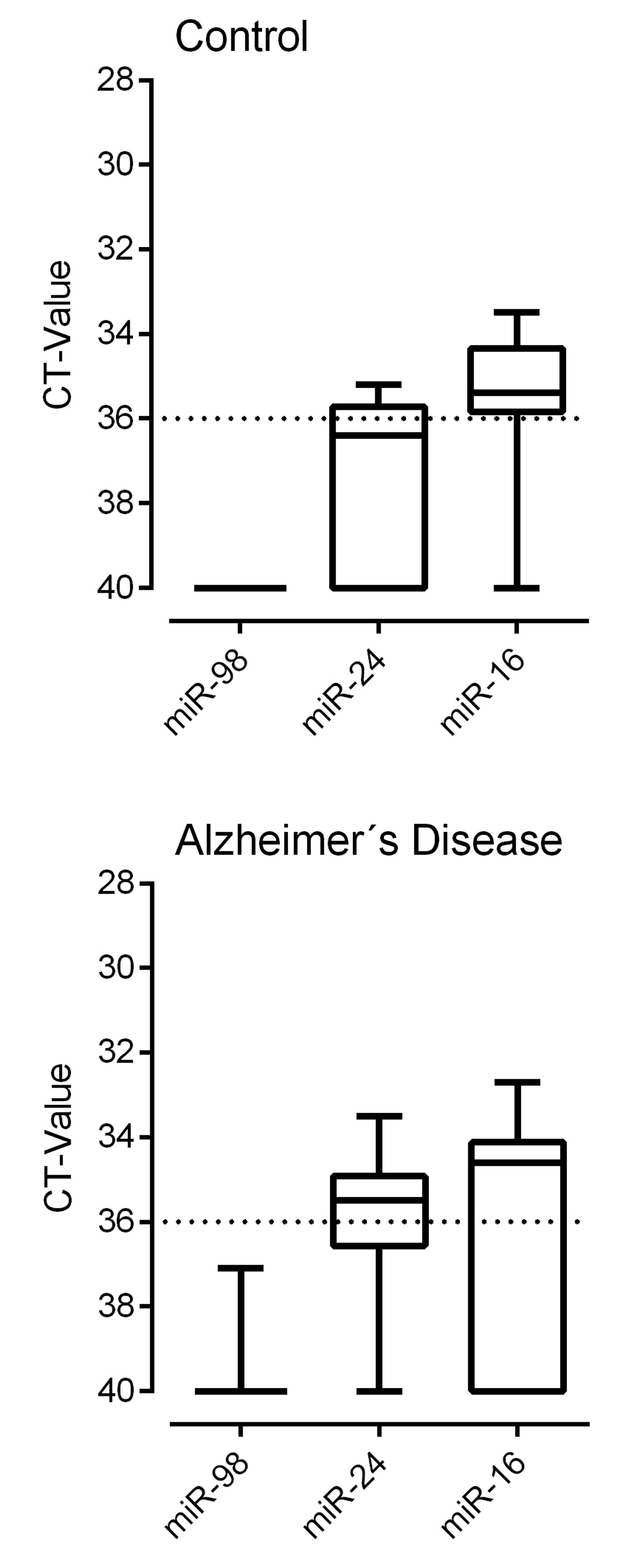

Supplement: S1 Fig — CSF samples from healthy controls (n = 9, Co-2 –Co-10) or patients with AD (n = 10, patient AD-3 –AD-12) were assayed by qPCR using primers specific for miR-98, miR-24, or miR-16. Data are presented as box-and-whisker-plots of mean CT-values performed in triplicates with median for each miRNA. (TIF) [file pone.0200602.s001.tif]

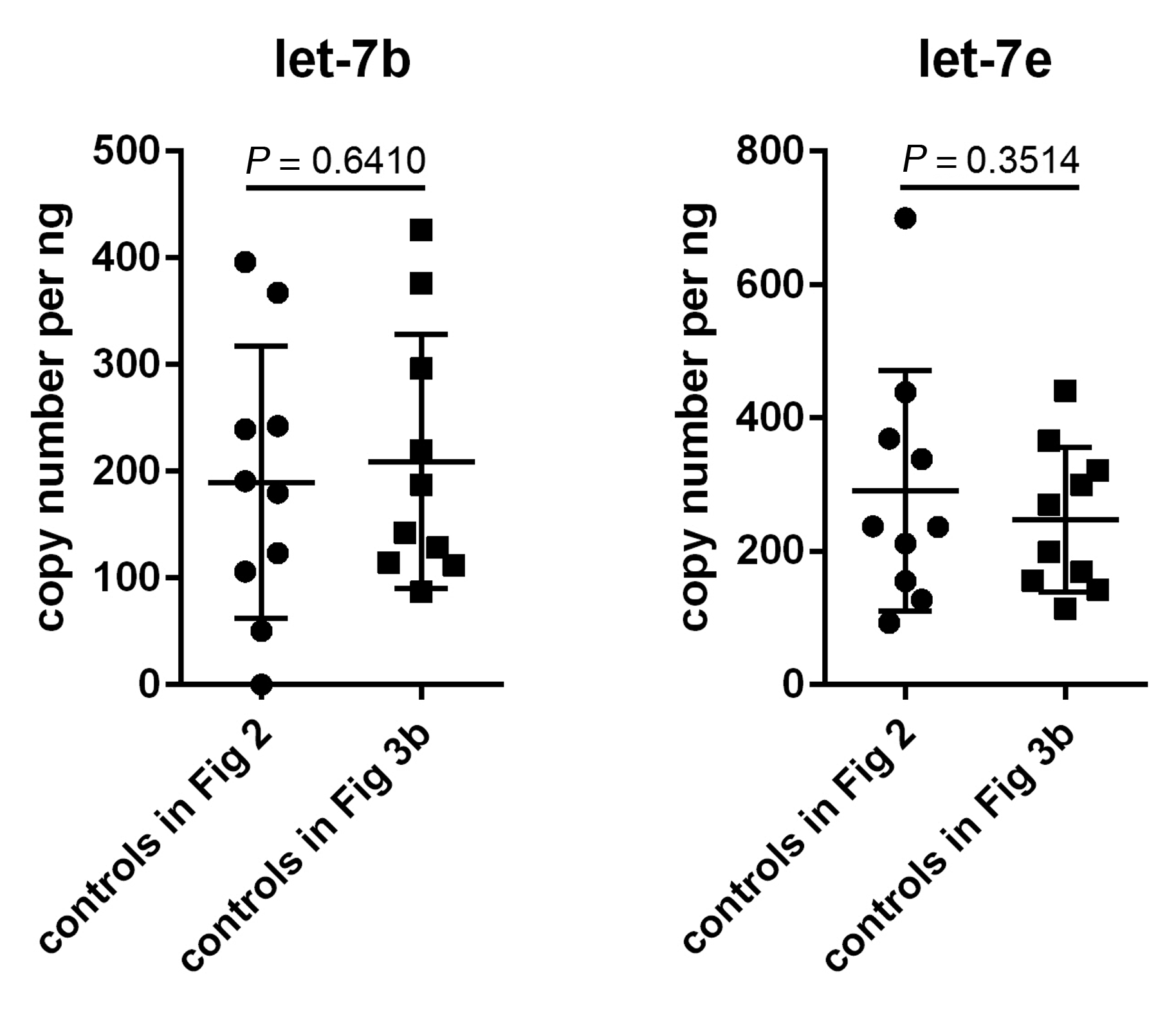

Supplement: S4 Fig — CSF from healthy controls depicted in Fig 2 and Fig 3B (n = 10, Co-1 –Co-10) were assayed by qPCR using primers specific for let-7b or let-7e and were normalized to the standard of the respective synthetic miRNA. Data are presented as mean ± SD. Each patient resembles one dot. Statistical analysis was performed using paired Student’s t-test. (TIF) [file pone.0200602.s004.tif]

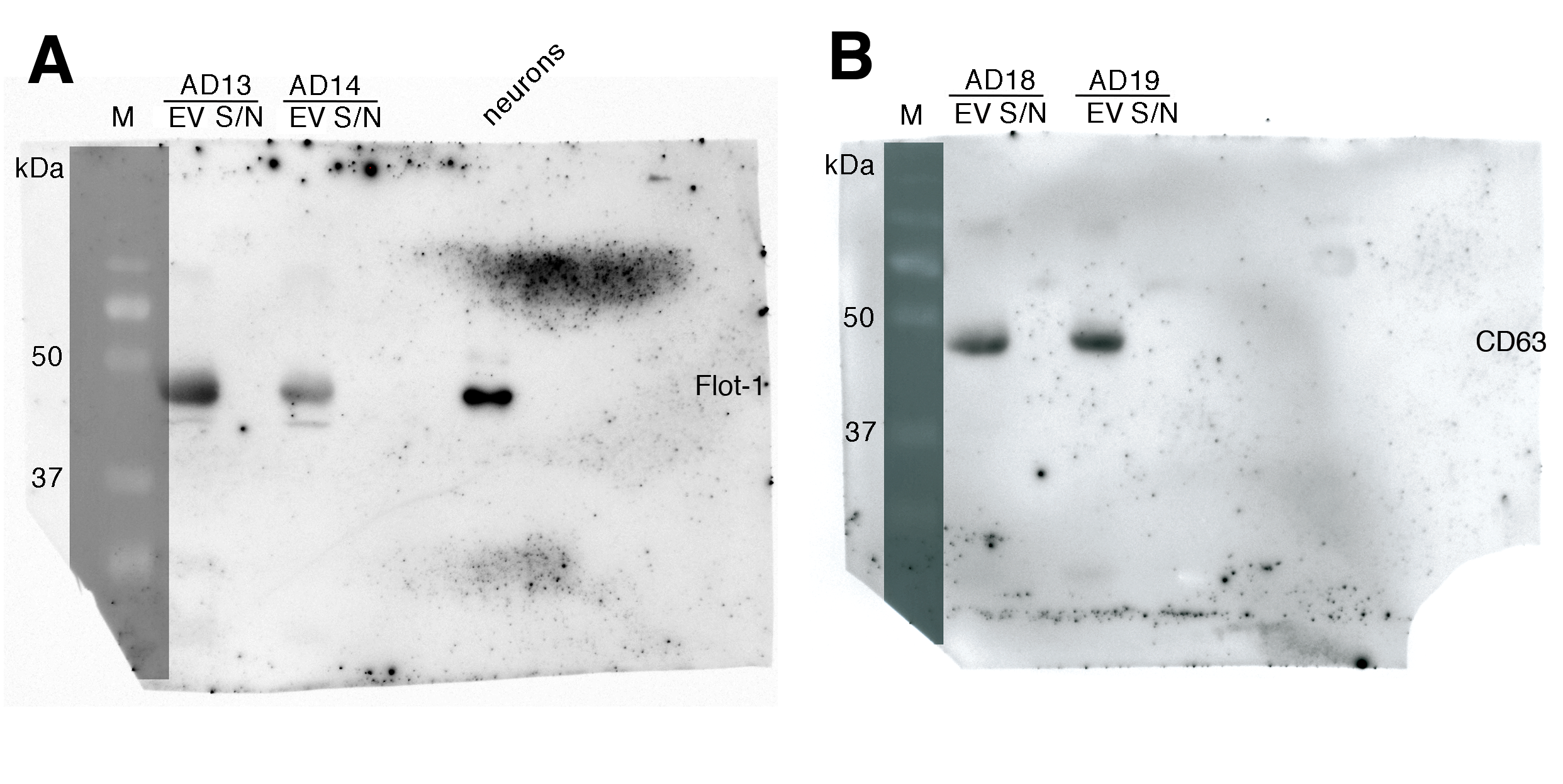

Supplement: S5 Fig — Extracellular vesicles (EV) were isolated from CSF of AD patients (n = 4), and both pellet of EV and supernatant (S/N) obtained during the isolation procedure were subjected to western blot using flotillin-1 (A) or CD63 (B) Abs. Neurons: Cortical neurons isolated from C57BL/6J mice. (TIF) [file pone.0200602.s005.tif]

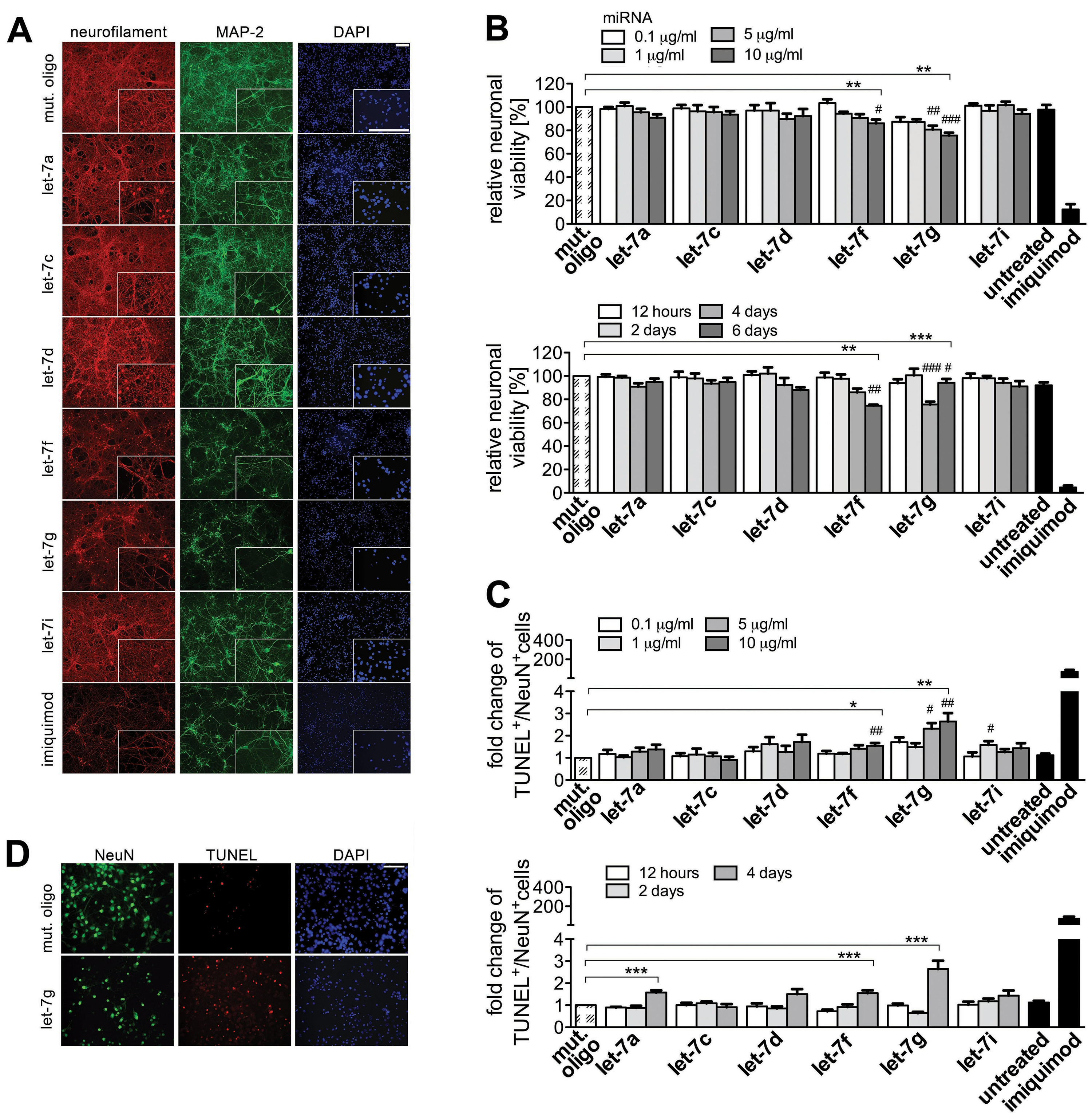

Supplement: S6 Fig — (A) Neurons from C57BL/6 mice were incubated with 5 μg/ml of the respective oligoribonucleotide or with 5 μg/ml imiquimod, which served as a positive control for TLR7-mediated neuronal cell death, for 5 days. Subsequently, cells were fixed and immunostained with neurofilament Ab (red), MAP-2 Ab (green), and stained with DAPI (blue). Scale bar 100 μm. (B, C) Neurons from C57BL/6 mice were incubated with indicated concentrations of let-7a, let-7c, let-7d, let-7f, let-7g, let-7i, or a mutated control oligoribonucleotide for 4 days, or with 10 μg/ml of the respective miRNA for indicated time points. 10 μg/ml imiquimod served as a positive control. Cells were immunostained with NeuN Ab, stained by TUNEL assay, or with DAPI. Each condition was performed in duplicate and averaged. NeuN-positive cells were quantified and were expressed as relative neuronal viability. TUNEL-positive cells were quantified, set in relation to NeuN-positive cells, and were expressed as fold-change. Mean ± SD from 3–4 individual experiments, Kruskal-Wallis test followed by Dunn´s Multiple comparison post hoc test of negative control vs. treatment. P values in (B) depicting the dose response: let-7f **p = 0.0033, let-7g **p = 0.0018; time response: let-7f **p = 0.0011, let-7g ***p = 0.0005. P values in (C) depicting the dose response: let-7f *p = 0.0223, let-7g **p = 0.0042; time response: let-7a ***p = 0.0008, let-7f ***p = 0.0009, let-7g ***p = 0.0005. #p<0.05, ##p<0.01, ###p<0.005. mut. oligo, mutated oligoribonucleotide. (D) Neurons were incubated with 10 μg/ml of the respective miRNA for 4 days. Subsequently, they were immunostained with NeuN Ab (green) and stained by TUNEL assay (red) and with DAPI (blue). Scale bar 50 μm. (TIF) [file pone.0200602.s006.tif]

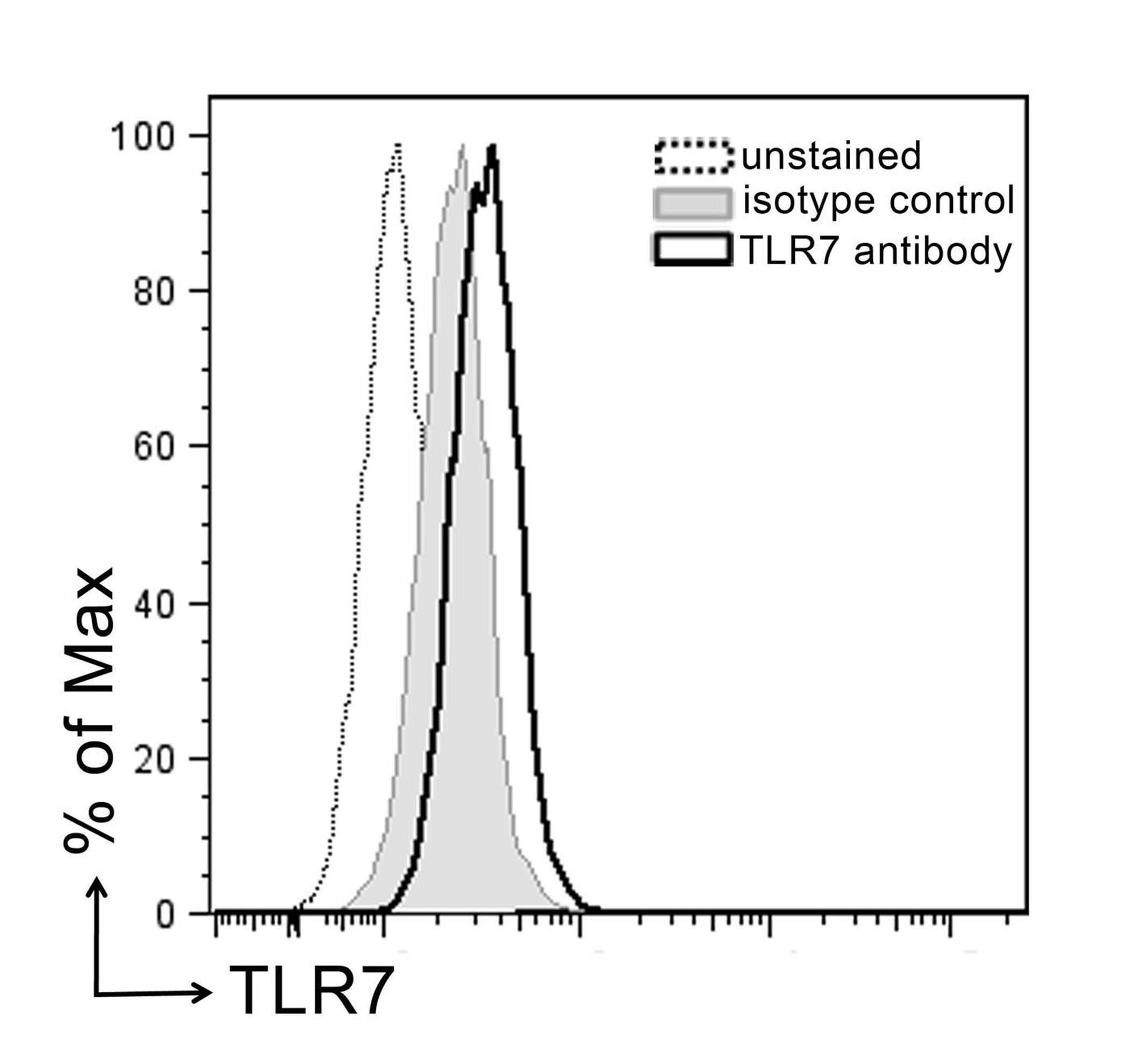

Supplement: S7 Fig — SH-SY5Y cells were fixed, permeabilized, stained with anti-TLR7 Alexa 488 Ab, and analyzed by flow cytometry. An Alexa488-conjugated isotype was used as a negative control. (TIF) [file pone.0200602.s007.tif]

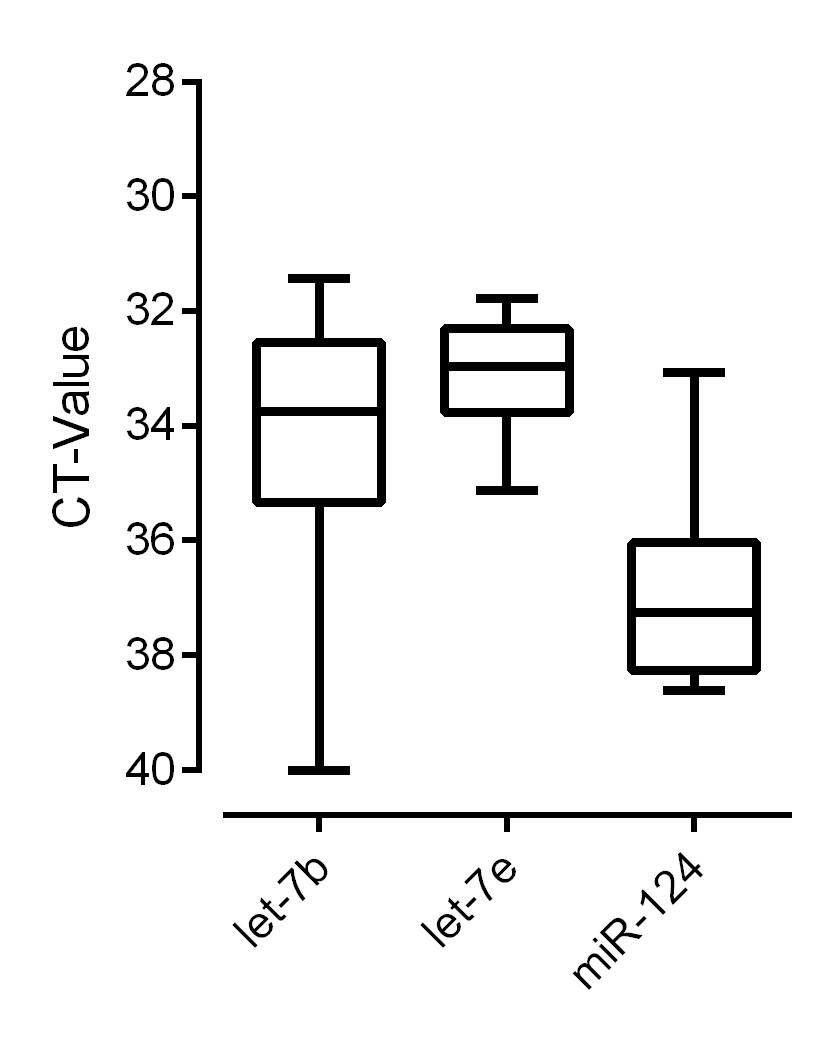

Supplement: S8 Fig — CSF samples from patients with AD (n = 10) that had been stored for 85,1+/-29,4 (mean ± SD) months after lumbar puncture at -80°C, were assayed by qPCR using primers specific for let-7b, let-7e, or miR-124. Data are presented as box-and-whisker-plots of mean CT-values performed in triplicates with median for each miRNA. (TIF) [file pone.0200602.s008.tif]
