# Supplementary material for: Distinct expression of the neurotoxic microRNA family let-7 in the cerebrospinal fluid of patients with Alzheimer's disease
Source: PLoS One. 2018 Jul 16;13(7):e0200602. doi: 10.1371/journal.pone.0200602 (PMC6047809; doi:10.1371/journal.pone.0200602)
Supplement: S2 Fig — CSF from patients with AD (n = 10–11, AD-1 –AD-6; AD-8 –AD-12) and healthy controls (n = 10, Co-1 –Co-10) were assayed by qPCR using primers specific for let-7a, let-7b, let-7c, let-7e, or let-7g and were normalized to the standard of the respective synthetic miRNA. Statistical analysis was performed using unpaired t-test, and p-values were adjusted for age and gender by ANCOVA. (DOCX) [file pone.0200602.s002.docx]

| **AD** | 891.65 **±** 370.25  (878.761 **±** 181.054) | 314.94 **±** 114.37  (288.422 **±** 48.605) | 294.26 **±** 271.08  (288.658 **±** 89.872) | 588.61 **±** 265.85  (633.032 **±** 84.665) | 16.76 **±** 12.12  (17.691 **±** 7.064) |
| --- | --- | --- | --- | --- | --- |
| **Control** | 905.77 **±** 484.80  (899.268 **±** 160.821) | 189.57 **±** 127.47  (202.740 **±** 44.777) | 211.25 **±** 130.57  (213.757 **±** 79.828) | 290.77 **±** 180.84  (229.744 **±** 77.996) | 20.76 **±** 20.11  (18.420 **±** 6.275) |
| ***t*-test**  **p-value** | 0.9425 | 0.0282 | 0.3944 | 0.0078 | 0.6984 |
| **adjusted**  **p-value** | 0.940 | 0.264 | 0.584 | 0.006 | 0.946 |

**let-7g**

**MW ± SD**

**(MW_adj_ ± SD_adj_)**

**let-7e**

**MW ± SD**

**(MW_adj_ ± SD_adj_)**

**let-7a**

**MW ± SD**

**(MW_adj_ ± SD_adj_)**

**let-7c**

**MW ± SD**

**(MW_adj_ ± SD_adj_)**

**let-7b**

**MW ± SD**

**(MW_adj_ ± SD_adj_)**

**let-7e**

**MW ± SD**

**(MW_adj_ ± SD_adj_)**

**let-7b**

**MW ± SD**

**(MW_adj_ ± SD_adj_)**

| **MDE** | 411.78 **±** 259.4763  (400.869 **±** 72.228) | 552.58 **±** 365.5791  (557.993 **±** 94.341) |
| --- | --- | --- |
| **Control** | 209.02 **±** 119.01  (226.589 **±** 67.242) | 247.80 **±** 108.32  (238.409 **±** 87.829) |
| **ANOVA**  **post hoc test**  **p-value** | < 0.05 | < 0.05 |
| **adjusted**  **p-value** | 0.109 | 0.031 |
