# Supplementary material for: Distinct expression of the neurotoxic microRNA family let-7 in the cerebrospinal fluid of patients with Alzheimer's disease
Source: PLoS One. 2018 Jul 16;13(7):e0200602. doi: 10.1371/journal.pone.0200602 (PMC6047809; doi:10.1371/journal.pone.0200602)
Supplement: S3 Fig — CSF from healthy controls (n = 10, Co-1 –Co-10) and from patients with FTLD (n = 8, FTLD-1 –FTLD-8) or MDE (n = 8, D-1 –D-8) were assayed by qPCR using primers specific for let-7b or let-7e and were normalized to the standard of the respective synthetic miRNA. Statistical analysis was performed using one-way ANOVA followed by Sidak’s multiple comparison post hoc test of the respective patient group vs. control group. p-values were adjusted for age and gender by ANCOVA. (DOCX) [file pone.0200602.s003.docx]

| **FTLD** | 144.33 **±** 55.96  (135.785 **±** 35.062) | 245.66 **±** 95.39  (256.828 **±** 36.371) |
| --- | --- | --- |
| **Control** | 209.02 **±** 119.01  (214.409 **±** 32.147) | 247.80 **±** 108.32  (239.933 **±** 33.348) |
| **ANOVA**  **post hoc test**  **p-value** | not significant | not significant |
| **adjusted**  **p-value** | 0.130 | 0.744 |

**let-7b**

**MW ± SD**

**(MW_adj_ ± SD_adj_)**

**let-7e**

**MW ± SD**

**(MW_adj_ ± SD_adj_)**

**let-7e**

**MW ± SD**

**(MW_adj_ ± SD_adj_)**

**let-7b**

**MW ± SD**

**(MW_adj_ ± SD_adj_)**

| **MDE** | 411.78 **±** 259.4763  (400.869 **±** 72.228) | 552.58 **±** 365.5791  (557.993 **±** 94.341) |
| --- | --- | --- |
| **Control** | 209.02 **±** 119.01  (226.589 **±** 67.242) | 247.80 **±** 108.32  (238.409 **±** 87.829) |
| **ANOVA**  **post hoc test**  **p-value** | < 0.05 | < 0.05 |
| **adjusted**  **p-value** | 0.109 | 0.031 |
